# Antioxidant Properties of Zinc and Copper—Blood Zinc-to Copper-Ratio as a Marker of Cancer Risk BRCA1 Mutation Carriers

**DOI:** 10.3390/antiox13070841

**Published:** 2024-07-14

**Authors:** Milena Matuszczak, Adam Kiljańczyk, Wojciech Marciniak, Róża Derkacz, Klaudia Stempa, Piotr Baszuk, Marta Bryśkiewicz, Cezary Cybulski, Tadeusz Dębniak, Jacek Gronwald, Tomasz Huzarski, Marcin Lener, Anna Jakubowska, Marek Szwiec, Małgorzata Stawicka-Niełacna, Dariusz Godlewski, Artur Prusaczyk, Andrzej Jasiewicz, Tomasz Kluz, Joanna Tomiczek-Szwiec, Ewa Kilar-Kobierzycka, Monika Siołek, Rafał Wiśniowski, Renata Posmyk, Joanna Jarkiewicz-Tretyn, Rodney Scott, Jan Lubiński

**Affiliations:** 1 Department of Genetics and Pathology, International Hereditary Cancer Center, Pomeranian Medical University, ul. Unii Lubelskiej 1, 71-252 Szczecin, Poland; milena.matuszczak@pum.edu.pl (M.M.); adam.kiljanczyk@pum.edu.pl (A.K.); klaudia.stempa@pum.edu.pl (K.S.); piotr.baszuk@pum.edu.pl (P.B.); marta.bryskiewicz@pum.edu.pl (M.B.); cezary.cybulski@pum.edu.pl (C.C.); tadeusz.debniak@pum.edu.pl (T.D.); jacek.gronwald@pum.edu.pl (J.G.); tomasz.huzarski@pum.edu.pl (T.H.); marcin.lener@pum.edu.pl (M.L.); anna.jakubowska@pum.edu.pl (A.J.); 2 Read-Gene, Grzepnica, ul. Alabastrowa 8, 72-003 Dobra, Poland; wojciech.marciniak@read-gene.com (W.M.); roza.derkacz@read-gene.com (R.D.); 3 Department of Clinical Genetics and Pathology, University of Zielona Góra, ul. Zyty 28, 65-046 Zielona Góra, Poland; gosiastawicka33@gmail.com; 4 Independent Laboratory of Molecular Biology and Genetic Diagnostics, Pomeranian Medical University in Szczecin, 70-204 Szczecin, Poland; 5 Department of Surgery and Oncology, University of Zielona Góra, Zyty 28, 65-046 Zielona Góra, Poland; szwiec72@gmail.com; 6 OPEN, Kazimierza Wielkiego 24 Str., 61-863 Poznań, Poland; godlewski.open@wp.pl; 7 Medical and Diagnostic Center, 08-110 Siedlce, Poland; artur.prusaczyk@centrum.med.pl; 8 Genetic Counseling Center, Subcarpatian Oncological Hospital, 18 Bielawskiego St, 36-200 Brzozów, Poland; ajasiewicz@yahoo.com; 9 Department of Gynecology, Gynecology Oncology and Obstetrics, Institute of Medical Sciences, Medical College, Rzeszow University, Rejtana 16c, 35-959 Rzeszow, Poland; jtkluz@interia.pl; 10 Department of Histology, Department of Biology and Genetics, Faculty of Medicine, University of Opole, 45-040 Opole, Poland; tomiczek.onk@gmail.com; 11 Department of Oncology, District Specialist Hospital, Leśna 27-29 St, 58-100 Świdnica, Poland; ewakilar@post.pl; 12 Holycross Cancer Center, Artwińskiego 3 St, 25-734 Kielce, Poland; monika.siolek@wp.pl; 13 Regional Oncology Hospital, Wyzwolenia 18 St, 43-300 Bielsko Biała, Poland; wiraf@poczta.onet.pl; 14 Department of Clinical Genetics, Medical University in Bialystok, 15-089 Bialystok, Poland; rposmyk@gmail.com; 15 Non-Public Health Care Centre, Cancer Genetics Laboratory, 87-100 Toruń, Poland; jarkiewicztretyn@poczta.onet.pl; 16 Medical Genetics, Hunter Medical Research Institute, Priority Research Centre for Cancer Research, Innovation and Translation, School of Biomedical Sciences and Pharmacy, Faculty of Health and Medicine, University of Newcastle; Pathology North, John Hunter Hospital, King and Auckland Streets, Newcastle, NSW 2300, Australia; rodney.scott@newcastle.edu.au

**Keywords:** zinc, copper, blood levels, BRCA1, BRCA1 mutation, germline mutation, cancer risk, breast cancer, ovarian cancer, propective cohort, modifiers

## Abstract

Pathogenic mutations in BRCA1 (BReast CAncer gene 1) confer high risks of both breast (up to 70%) and ovarian (up to 40%) cancers. Zinc (Zn) and copper (Cu) are essential for various physiological functions, including antioxidant reactions. Their balance, reflected in the Zn/Cu ratio, plays a crucial role in maintaining redox homeostasis, which is vital for cancer prevention. This study examines the antioxidant properties of Zn and Cu, specifically focusing on the blood Zn/Cu ratio as a potential marker for cancer risk among BRCA1 mutation carriers. The study cohort consisted of 989 initially unaffected women, followed up for 7.5 years. Blood samples were analyzed using inductively coupled plasma mass spectrometry. Although individual Zn and Cu levels did not significantly correlate with overall cancer risk, those women with a Zn/Cu ratio above 6.38 experienced a significantly lower cancer risk than women with a ratio below this cut-off point. This suggests that the Zn/Cu ratio may be a valuable biomarker for cancer prevention in this high-risk group. Given the increased cancer risk in BRCA1 mutation carriers, optimizing Zn and Cu levels through dietary and active interventions could provide a preventive strategy.

## 1. Introduction

The BRCA1 gene plays a critical role in various physiological processes, including the cell cycle and gene transcription [1] regulation, apoptosis [2], repair of double-strand DNA breaks (DSBs), maintaining genomic stability, and protecting, repairing, and restarting stalled and damaged replication forks (independently of DSB repair) [3]. Additionally, BRCA1 protects stressed replication forks from degradation by stabilizing RAD51 nucleofilaments [4].

Tumors in patients with BRCA1 mutations typically exhibit a loss of heterozygosity (LOH) or other inactivating somatic alterations of the wild-type copy [5]. Mutations in the BRCA1 gene often affect one of the two essential domains, RING or BRCT, necessary for homologous recombination (HR), causing HR deficiency. This disrupts the repair of DSBs, leading to genomic instability, which drives oncogenesis and cancer proliferation. BRCA1-mutated tumors rely on alternative repair mechanisms due to impaired HR [6].

Women with BRCA1 mutations have an increased lifetime risk of various cancers, including breast cancer (up to 70%) and ovarian cancer (up to 40%) [7]. Additionally, there is an increased risk of developing a second primary breast cancer within 10 and 20 years by 20–30% and 40–50%, respectively [7]. The risk for esophageal, pancreatic, and stomach cancers is also approximately three times higher compared to the general population (RR = 2.9, RR = 3.34, and RR = 3.69, respectively) [8,9,10]. An increased risk of colorectal cancer before age 50 has also been observed [11].

Further understanding of the underlying mechanisms of cancer development among BRCA1 carriers may provide valuable insights into modifiable determinants and prevention strategies.

Besides the influence of inherited mutations on the incidence of cancer, women with a pathogenic BRCA1 mutation have various non-genetic modifiable factors. These factors include age, family history, breastfeeding [12], prophylactic procedures (salpingo-oophorectomy, mastectomy [13], and tubal ligation [12,14]), hormone (such as contraceptives) [12,15] or replacement therapy, tamoxifen [16], late age at menarche [17] and menopause [18], fertility treatment [19], exposure to radiation, chronic infections and inflammations [20], and lifestyle habits (like alcohol, coffee consumption [21], smoking, physical activity, dietary energy intake [22], BMI [23], and dietary components (variety of vegetables and fruits [24], including elements [25,26,27])).

Among the elements, we can distinguish zinc and copper. Zinc’s anticancer properties are generally attributed to its antioxidant capabilities and its role in shielding cells from oxidative stress. It plays a crucial role in regulating essential processes involved in cancer progression, such as DNA repair, gene expression, and programmed cell death [27]. Copper remains vital for cellular metabolism, participating in various physiological functions, including antioxidant reactions and mitochondrial respiratory chain activity. In the discussion of this paper, we further describe the antioxidant properties of zinc and copper and the potentially harmful effects of deficiency and excess, which may be involved in tumorigenesis. The zinc-to-copper ratio is associated with oxidative stress, inflammation, and hormones [28,29]. In addition, the ratio of zinc to copper correlates with the modulation of immune defense, growth stimuli, and a response to stress. The Zn/Cu ratio is also presumed to be associated with the body’s repair attempts in old age [29]. The levels of Zn and Cu are tightly controlled by compensatory mechanisms designed to maintain their stability within specific ranges of nutritional intake. Studies published to date indicate that a form of redox balance exists between zinc and copper, which is reflected in the ratio of these elements.

The study of zinc and copper ratios in the blood has been ongoing for over 50 years [30]. Previous research has evaluated blood/serum levels of zinc and copper in a prospective manner concerning cancer risk [31,32] and survival [33,34]. Prospective studies on cancer risk using zinc and copper status and their ratio in the blood are sparse in current literature. In the literature, we identified three prospective studies that measured blood levels of zinc and copper and their ratio to evaluate cancer risk. All of these studies [31,32,35] were case-control studies conducted within a large prospective multicenter cohort (EPIC), with one specifically investigating breast cancer risk [35]. To the best of our knowledge, there are no prospective studies examining the BRCA1 Zn/Cu ratio and its association with cancer risk. This gap in research has encouraged us to conduct a prospective study on BRCA1 mutation carriers to determine if the Zn/Cu ratio can be a useful preventive biomarker in this population.

## 2. Materials and Methods

The study subjects were 989 initially unaffected adult women who received genetic counseling and testing between 2011 and 2017 at the Clinical Hospitals of the Pomeranian Medical University in Szczecin, Poland, or at associated hospitals or outpatient clinics. Patients were seen at these facilities for the period specified and were enrolled in the study. At the first study visit, a fasting blood sample was collected from each study participant to be used for genetic testing for *BRCA1* mutations. For analysis, 10 mL of peripheral blood was collected into a vacutainer tube containing ethylenediaminetetraacetic acid (EDTA) from all study participants. All blood samples were collected between 8 a.m. and 2 p.m. and stored at −80 ℃ until analysis.

Participants were included in this study if a deleterious *BRCA1* variant was detected. Medical charts were reviewed for date of diagnosis, age at enrollment (<50/≥50), ovary removal (yes/no), smoking status (ever/never), contraceptive use (ever/never), diabetes (yes/no), dietary supplements (ever/never), hormonal therapy (ever/never), and BMI (<18.5/18.5–24.9/25.0–29.9/≥30.0).

This study was conducted in accordance with the Helsinki Declaration and with the consent of the Ethics Committee of the Pomeranian Medical University in Szczecin under the number KB-0012/73/10 on 21 June 2010. All participants provided written informed consent.

### 2.1. Measurement of Blood Zinc and Copper Level

The blood samples were obtained from fasting individuals through venipuncture using the Vacutainer^®^ System (BD #368381, Plymouth, UK). Blood was carefully divided into new cryovials and then frozen at −80 °C until analysis.

The elemental composition of the samples was determined using the inductively coupled plasma mass spectrometry (ICP-MS) technique with the NexION 350D instrument (PerkinElmer, Norfolk, VA, USA). The KED (Kinetic Energy Discrimination) mode was employed for element determination, and rhodium was used as an internal standard to compensate for instrument drift and matrix effects. Detailed information regarding the specific parameters of the NexION 350D instrument used in the measurements can be provided upon request. During analysis, the blood samples were diluted 40-fold with a blank reagent (70 µL of blood: 2730 µL of buffer).

The blank reagent used consisted of high-purity water (>18 MΩ), TMAH (AlfaAesar, Kandel, Germany), Triton X-100 (PerkinElmer, Shelton, CT, USA), EDTA (Merck, Darmstadt, Germany), and ethyl alcohol (Merck, Darmstadt, Germany).

Calibration curve standards were prepared by diluting the stock solution of 1000 µg/mL zinc and copper Standard (PerkinElmer Pure Plus, Shelton, CT, USA) with the blank reagent. The calibration method used was matrix-matched, and the correlation coefficients for the calibration curve were always greater than 0.999.

The accuracy and precision of the measurements were evaluated using certified reference materials (CRM): ClinChek^®^ Plasmonorm Whole Blood Level 1 (Recipe, Munich, Germany) and Seronorm Whole Blood Level 2 (Sero, Norway). Technical details, plasma operating settings, and mass spectrometer acquisition parameters can be provided upon request. The testing laboratory participates in an independent external quality assessment scheme, QMEQAS (Quebec Multielement External Quality Assessment Scheme), organized by the Institut National de Santé Publique du Québec.

### 2.2. Statistical Analysis

All study participants were assigned to one of three categories (tertiles) depending on their zinc and copper levels. Patients were followed from the date of blood draw until the development of cancer or the date of the last follow-up. To estimate the hazard ratios (HRs) for cancer risk according to the zinc and copper tertile, univariable and multivariable Cox proportional hazards regression analysis was performed. The reference levels for zinc and copper were taken as the lowest tertile. In the multivariable models, the following variables were taken into account: zinc and copper level (tertile), age at blood draw (<50 vs. ≥50), oral contraceptive use (yes/no), hormone replacement therapy use (yes/no), smoking history (yes/no), oophorectomy (yes/no), and BMI (<18.5/18.5–24.9/25.0–29.9/≥30.0). Due to missing data, 84 patients were excluded from the statistical analysis.

All statistical analyses were performed using the R statistical environment (R: A Language and Environment for Statistical Computing, Vienna, Austria 2023).

## 3. Results

The study group consisted of 989 women with a *BRCA1* mutation. Among our cohort, there were 559 women with c.5266dupC, n = 250 with c.181T/G, n = 50 with c.4035delA, n = 18 with c.3700_3704delGTAAA, n = 14 with c.1687C > T, n = 12 with c.5251C > T, n = 9 with c.66_67delAG (c.68_69delAG), and n = 9 with c.676delT; 68 women had other rarer mutations. The women were unaffected at the time of inclusion in the study. The mean age of enrollment (blood draw) was 44.0 years. The patients were followed up for an average of 7.52 years, during which time 173 new cancers occurred in various organs (121 cases of breast cancers, 29 cases of ovarian cancers, and 23 cancers at other sites). The characteristics of the study group are shown in Table 1.

A total of 573 of the women had a risk-reducing oophorectomy at a mean age of 45.2 years. A total of 204 of the women had the oophorectomy prior to the blood draw and 369 women had the oophorectomy during the follow-up period.

In the study group, we compared the results of zinc, copper, and the zinc-to-copper ratio using the calculated cut-off point (Figure S1 Supplementary Materials).

The associations between blood zinc levels and cancer risk in BRCA1 mutation carriers, which we described in an earlier publication [27], did not show a statistically significant reduction in the risk of any of the cancers; we observed tendencies, although not statistically significant, for ovarian and breast cancers.

Similarly, outcomes of the analysis of copper levels in the blood showed a tendency to reduce the risk of cancer with decreasing copper values (<863.33). However, the obtained results were not statistically significant (Tables S1–S3 in Supplementary Materials).

**Figure 1:**
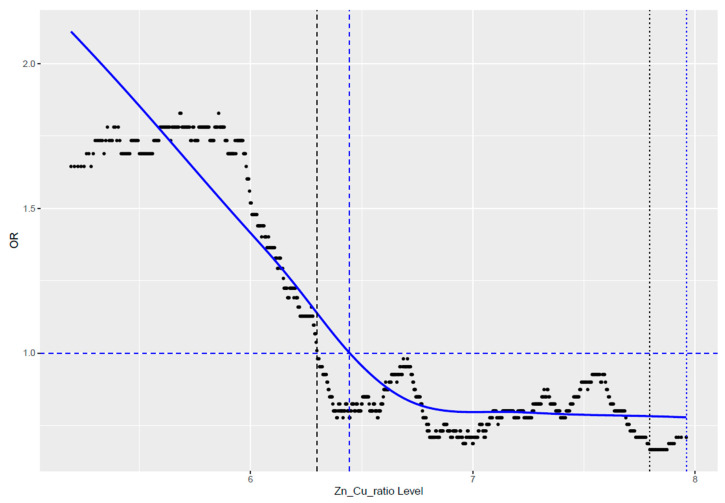
Correlation of cancer risk with the ratio value of zinc-to-copper.

We observed that the value of their ratio after crossing a certain value became harmful (Figure 1). Our observation showed that in the studied cohort of women, this established cut-off point was 6.38. Values below this cut-off point were unfavorable and were associated with an increased risk of cancer.

When we divided the group by using a cut-off point = 6.38, we achieved an almost 1.5-fold statistically significant reduction in the risk of any cancer (HR = 1.48; CI = 1.07–2.04; *p* = 0.018) (Table 2).

The tendency, although not statistically significant, was also present for breast cancer and was associated with a more than 1.3-fold increased risk of breast cancer in the group of women who had a Zn/Cu ratio value of less than 6.38 (HR = 1.31; CI = 0.90–1.93; *p* = 0.2) (Table S4 in Supplementary Material).

The most pronounced, more than 1.75-fold, reduction of cancer risk was observed in ovarian cancer, but this result was not statistically significant (HR = 1.78; CI = 0.77–4.11; *p* = 0.2) (Table S5 in Supplementary Material).

## 4. Discussion

Essential elements play a crucial role in maintaining the body’s normal functions, including anti-cancer protective mechanisms. Zinc and copper have been recognized as influential in the occurrence of cancer. Understanding these influences is particularly important for individuals with a heightened risk of cancer, such as BRCA1 mutation carriers, who face a significantly increased lifetime risk of breast (up to 70%) and ovarian (up to 40%) cancer. Thus, gaining insights into the factors influencing the remaining 30% and 60% risks, respectively, for these malignancies is crucial.

Our earlier research, despite not demonstrating statistically significant reductions in cancer risk associated with blood zinc levels in BRCA1 mutation carriers, indicated trends [27]. Given zinc’s recognized antioxidant effects and these observed trends, we conducted further analyses, incorporating additional factors.

Zinc is crucial for maintaining DNA integrity and immune system function, as well as possessing antioxidant properties that help reduce oxidative stress [36]. Its role in inhibiting cell proliferation and inducing apoptosis in cancer cells makes it a valuable component of cancer prevention strategies. Zinc is an essential cofactor for many proteins involved in DNA repair, and its optimal level reduces the risk of further DNA damage accumulation. Zinc affects the activity of enzymes and transcription factors regulating the cell cycle and the function of proteins such as p53, which helps regulate the cell cycle and prevent uncontrolled growth of cancer cells. It also stabilizes chromatin elements and DNA repair proteins, which is key to maintaining genomic stability. Zinc protects cells from damage caused by reactive oxygen species (ROS), reducing oxidative stress and decreasing the risk of cancer development. Additionally, it is essential for the function of pro-apoptotic proteins, which helps eliminate damaged cells that could lead to cancer formation. Studies show that zinc supplementation has chemopreventive effects in the case of cancers. This might be due to reduced inflammation and oxidative stress in patients who supplement zinc [37,38,39].

The results obtained in our study showed that there is a tendency (not statistically significant) of a potential benefit of maintaining low (<863.33) copper levels in BRCA1 mutation carriers. Copper is an essential element, but like a double-edged sword, both elevated and decreased copper levels can pose risks, potentially exacerbating cancer development. Copper showcases both advantageous (antioxidant) and harmful (pro-oxidant) effects on cellular processes.

Copper exhibits several antioxidant properties critical for maintaining cellular health. Firstly, copper serves as an essential cofactor for superoxide dismutase (SOD), an enzyme that catalyzes the conversion of superoxide radicals into oxygen and hydrogen peroxide, thereby reducing oxidative stress in cells [40]. Additionally, copper is necessary for the synthesis of ceruloplasmin, a protein that not only scavenges free radicals but also facilitates the transport of copper in the bloodstream [41].

Furthermore, copper participates in antioxidant pathways that protect lipids from peroxidation, a harmful process where free radicals attack lipid molecules in cell membranes [42]. Another crucial function of copper is its role in maintaining the balance between oxidized and reduced forms of molecules within cells, which is essential for proper cellular function and protection against oxidative damage [43]. Copper is a component of ubiquitous and multifunctional metalloenzymes that facilitate the reduction of molecular oxygen, a process vital for various physiological functions, as indicated in references [44,45]. These antioxidant properties underscore copper’s significance in protecting cells from oxidative damage. The accumulation of copper in cancer cells manifests in two distinct biological traits. On one hand, it can stimulate tumor development by promoting cell proliferation, metastasis, and angiogenesis. On the other hand, it can also induce programmed cell death in tumor cells, thereby impeding tumor progression. In recent years, the terms “cuproplasia” and “cuproptosis” have emerged, explaining to some extent the complex mechanisms of copper involvement in tumorigenesis [39]. Cuproptosis involves mitochondrial copper directly attaching to proteins, leading to post-translational modifications such as lipoylation. This process, through a complex interaction associated with proteotoxic stress, leads to cell death. There is no research evidence to suggest that these processes do not occur in BRCA1 mutation carriers.

Copper plays a crucial role in the anticancer and preventive mechanisms by inhibiting angiogenesis, which is the formation of new blood vessels that tumors rely on for growth [46]. This inhibition is achieved by reducing angiogenic factors like VEGF and FGF. Additionally, copper promotes apoptosis in cancer cells by disrupting copper-dependent enzymes, leading to cell death. By targeting metalloproteins and inhibiting copper-dependent enzymes like LOX [47] and SOD1, copper hinders processes like extracellular matrix remodeling [48]. It also modulates the immune system [49], enhancing its ability to recognize and destroy cancer cells. In addition, copper can impact epigenetic modifications, potentially restoring the expression of tumor suppressor genes and silencing oncogenes. It interferes with the tumor microenvironment by inhibiting Matrix metalloproteinases (MMPs), which are involved in extracellular matrix degradation. Copper inhibits oncogenic pathways like PI3K/Akt and MAPK, which are crucial for cancer cell proliferation.

An imbalance in copper and zinc levels (elevated zinc and/or reduced copper) hinders the antioxidant function of various enzymes [50]. Prolonged oxidative stress could heighten the likelihood of developing breast cancer and impact the initial phases of cancer development and advancement [51,52].

The copper-to-zinc ratio (Cu/Zn ratio) is believed to serve as a more precise predictive indicator compared to individual copper or zinc levels [53]. An imbalance between copper and zinc levels can lead to the overproduction of ROS, which in turn promotes tumorigenesis [54]. Given the sensitivity of mitochondrial DNA to ROS, there may exist a theoretical link between copper and zinc levels and mutations in mitochondrial DNA in cancer cells [55]. A recent comprehensive analysis determined that an elevated Cu/Zn ratio correlates with a heightened risk of breast cancer [56].

The interactions between zinc and copper within the human body are intricate, beginning in the small intestine, where the intake and status of each element can influence the absorption of the other [57]. They are crucial components of copper–zinc SOD (CuZnSOD), pivotal for maintaining DNA stability and integrity to mitigate cancer development.

The literature often highlights the beneficial potential of maintaining an optimal Zn/Cu ratio. However, prospective studies focusing on this ratio in cancer risk are limited. Studies by Stepien et al., based on the European Prospective Investigation into Cancer and Nutrition (EPIC) cohort, revealed significant associations between the Zn/Cu ratio and colorectal cancer [32] and hepatocellular carcinoma risk [31].

The literature’s rationale prompted us to investigate the Zn/Cu ratio in our cohort. We observed interdependence between zinc and copper levels, with low Zn often coinciding with Cu deficiency and vice versa. By establishing a cut-off point of 6.38 for the Zn/Cu ratio, we found that ratios below this significantly increased cancer risk, while values equal to or greater than 6.38 represented an optimal range with relatively lower risks.

There is a lack of information in the literature on how catalysts (including SOD1) differ in BRCA1 mutation carriers compared to the general population. However, since the observed correlations between cancer risk and Zn/Cu ratio are similar between the general population and our cohort, we can assume that these mechanisms described above can also be valuable in BRCA1 carriers.

The tumor suppressor gene BRCA1 and its encoded proteins are vital for preserving the integrity of the genome, playing a crucial role in repairing DNA double-strand breaks through homologous recombination [58]. The primary model for cancer development linked to BRCA1 mutations is based on the “two-hit” hypothesis, where one allele is lost, and the other undergoes a loss-of-function mutation [59]. This mechanism forms the basis of the molecular pathogenesis of BRCA1-related cancers, with key factors including genomic instability and oxidative stress [60]. Genomic instability arises from deficiencies in DNA repair caused by BRCA1 mutations, particularly affecting homologous recombination, leading to the accumulation of DNA damage and an increased risk of oncogenic mutations [61]. Additionally, the loss of BRCA1 function is associated with chromosomal aberrations like breaks, fusions, and translocations, further destabilizing the genome [58]. Cells with BRCA1 mutations often exhibit LOH, where the normal BRCA1 allele is lost, resulting in the complete loss of BRCA1 function in these cells [62]. Oxidative stress poses a threat to genomic stability, with BRCA1 playing a crucial role in regulating oxidative stress as a defense mechanism in individuals without pathogenic variants. Mutation carriers are more vulnerable to the effects of ROS and the resulting oxidative DNA damage, contributing to the development of cancer [63]. BRCA1 indirectly influences oxidative stress regulation through the p53 protein, which responds to ROS-induced oxidative stress. BRCA1 activates and stabilizes p53 [64], facilitating the p53-dependent transcription of genes involved in DNA repair. Mutations in BRCA1 disrupt these interactions, reducing the cell’s ability to effectively manage oxidative stress. Furthermore, BRCA1 is essential for cell cycle checkpoints, particularly in response to DNA damage [65]. Acting as an E3 ubiquitin ligase, BRCA1 polyubiquitinates G2/M cell cycle proteins, marking them for degradation and preventing their accumulation [66]. Mutations in BRCA1 allow cells with DNA damage to continue dividing, thereby increasing the risk of cancer development.

Considering these findings, addressing zinc deficiency through dietary changes or supplementation may be advantageous. It is advisable to avoid factors known to elevate copper levels, such as hormone replacement therapy and contraception [67].

Given our patients in the BRCA1 cohort, studies on the efficiency of active interventions aimed at improving the Zn/Cu ratio are strongly justified. While there are promising trials in the literature actively targeting copper reduction, such interventions specifically tailored to women with BRCA1 mutations are currently lacking.

Our study has several limitations that need to be acknowledged. Firstly, the number of BRCA1 mutation carriers included in the study was relatively small, which could limit the generalizability of the findings. Additionally, the patients were selected from cancer genetics outpatient clinics, introducing a potential selection bias as most women were referred due to a positive family history of cancer. This selection criterion may not represent the broader population of BRCA1 mutation carriers. Furthermore, the observation period could have been longer to capture more extended outcomes and trends. Another limitation is the homogeneity of the studied population, which was primarily of a Polish genetic background. This genetic uniformity may not reflect the diversity seen in broader, more varied populations. Therefore, validation studies on additional groups and in various populations are necessary to confirm the findings and ensure their applicability across different genetic backgrounds and demographic settings.

Hence, there is considerable promise in expanded efforts targeting BRCA1, necessitating broader cohort inclusion, active intervention implementation, and clinical trials focusing on prevention by optimizing the Zn/Cu ratio. Furthermore, it is crucial to recognize that this relationship can serve both as a marker and a potential target for intervention.

## 5. Conclusions

There is a need to consider optimization of the levels of both elements, Zn and Cu. It seems to be important in women at genetic risk (BRCA1 carriers) for cancers. The Zn/Cu ratio may be a significant biomarker of cancer risk in this cohort.

## Figures and Tables

**Table 1:** Group characteristics.

	N = 989
**Age at enrollment**	
<50 years	775 (78.36%)
≥50 years	214 (21.64%)
**Smoking**	
never	720 (72.80%)
ever	264 (26.69%)
missing data	5 (0.51%)
**Hormonal therapy**	
never	720 (72.80%)
ever	263 (26,59%)
missing data	6 (0.61%)
**Oophorectomy**	
no	413 (41,76%)
yes	576 (58,24%)
missing data	0 (0.00%)
**Oral Contraceptive use**	
never	501 (50,66%)
ever	481 (48,64%)
missing data	7 (0,70%)
**Diabetes**	
no	880 (88.98%)
yes	62 (6.27%)
missing data	47 (4.75%)
**Body Mass Index (kg/m^2^)**	
<18.5	56 (5.66%)
18.5–24.9	553 (55.92%)
25.0–29.9	237 (23.96%)
≥30.0	95 (9.61%)
missing data	48 (4.85%)
**Dietary supplements usage**	
never	500 (50.56%)
ever	489 (49.44%)
**New cancer site (n = 174) (by the first cancer)**	
breast	122 (70.11%)
ovarian	29 (16.67%)
bladder	2 (1.15%)
cervix	3 (1.72%)
colon	2 (1.15%)
kidney	1 (0.57%)
leukemia	2 (1.15%)
lung	3 (1.72%)
pancreas	1 (0.57%)
salivary gland	1 (0.57%)
sarcoma	1 (0.57%)
site unknown	1 (0.57%)
skin	1 (0.57%)
thyroid	3 (1.72%)
urothelial	1 (0.57%)
abdomen-CSU	1 (0.57%)

**Table 2:** Incidence of cancers with different organ localization in initially unaffected BRCA1 mutation carriers according to zinc-to-copper ratio values.

	New Cancer Frequency			New Cancer Univariable COX Regression			New Cancer Multivariable COX Regression		
Characteristic	Overall, N = 914 ^1^	0, N = 763 ^1^	1, N = 151 ^1^	HR ^2^	95% CI ^2^	*p*-Value	HR ^2^	95% CI ^2^	*p*-Value
**Zn/Cu ratio**									
II (reference): 6.38–16.03 (7.47)	540 (59%)	467 (61%)	73 (48%)	—	—		—	—	
I: 0.00–6.37 (5.26)	374 (41%)	296 (39%)	78 (52%)	1.53	1.11, 2.11	0.009	1.48	1.07, 2.05	0.017
**Year of birth**									
≤1965	233 (25%)	181 (24%)	52 (34%)	—	—		—	—	
>1985	136 (15%)	129 (17%)	7 (4.6%)	0.27	0.12, 0.59	0.001	0.27	0.09, 0.85	0.025
1965–1975	228 (25%)	188 (25%)	40 (26%)	0.79	0.52, 1.19	0.3	0.88	0.44, 1.78	0.7
1975–1985	317 (35%)	265 (35%)	52 (34%)	0.76	0.52, 1.11	0.2	0.80	0.33, 1.95	0.6
**Age of blood draw**									
≤40	548 (60%)	471 (62%)	77 (51%)	—	—		—	—	
>50	175 (19%)	135 (18%)	40 (26%)	1.57	1.07, 2.30	0.022	1.20	0.49, 2.95	0.7
40–50	191 (21%)	157 (21%)	34 (23%)	1.25	0.84, 1.88	0.3	1.00	0.54, 1.88	>0.9
**Oral contraception**									
no	454 (50%)	378 (50%)	76 (50%)	—	—		—	—	
yes	460 (50%)	385 (50%)	75 (50%)	0.97	0.70, 1.33	0.8	1.08	0.76, 1.53	0.7
**Hormonal replacement therapy**									
no	659 (72%)	547 (72%)	112 (74%)	—	—		—	—	
yes	255 (28%)	216 (28%)	39 (26%)	0.78	0.54, 1.12	0.2	0.68	0.46, 1.00	0.050
**Smoker**									
Never	517 (57%)	444 (58%)	73 (48%)	—	—		—	—	
Current	207 (23%)	168 (22%)	39 (26%)	1.39	0.94, 2.05	0.10	1.34	0.91, 1.99	0.14
Former	190 (21%)	151 (20%)	39 (26%)	1.48	1.00, 2.18	0.050	1.41	0.95, 2.08	0.087
**BMI**									
<18.5	539 (59%)	455 (60%)	84 (56%)	—	—		—	—	
18.5–25.0 (reference)	54 (5.9%)	44 (5.8%)	10 (6.6%)	1.18	0.61, 2.26	0.6	1.30	0.67, 2.52	0.4
≥30.0	92 (10%)	75 (9.8%)	17 (11%)	1.29	0.77, 2.18	0.3	1.04	0.60, 1.78	0.9
25.0–30.0	229 (25%)	189 (25%)	40 (26%)	1.12	0.77, 1.64	0.5	0.96	0.65, 1.43	0.8
**Adnexectomy**									
no	423 (46%)	331 (43%)	92 (61%)						
yes	491 (54%)	432 (57%)	59 (39%)						

^1^ n (%). ^2^ HR = Hazard Ratio, CI = Confidence Interval.

## Data Availability

The original contributions presented in the study are included in the article/Supplementary Material, further inquiries can be directed to the corresponding author/s.

## Supplementary Materials

The following supporting information can be downloaded at: https://www.mdpi.com/article/10.3390/antiox13070841/s1, Figure S1: Zinc and copper distribution in studied cohort; Table S1: Incidence of cancers with different organ localization in initially unaffected BRCA1 mutation carriers according to blood copper levels; Table S2: Incidence of breast cancers in initially unaffected BRCA1 mutation carriers according to blood zinc levels; Table S3: Incidence of ovarian cancers in initially unaffected BRCA1 mutation carriers according to copper ratio values; Table S4: Incidence of breast cancers in initially unaffected BRCA1 mutation carriers according to zinc to copper ratio values; Table S5: Incidence of ovarian cancers in initially unaffected BRCA1 mutation carriers according to zinc to copper ratio values.
