# Supplementary material for: Antioxidant Properties of Zinc and Copper—Blood Zinc-to Copper-Ratio as a Marker of Cancer Risk BRCA1 Mutation Carriers
Source: Antioxidants (Basel). 2024 Jul 14;13(7):841. doi: 10.3390/antiox13070841 (PMC11273827; doi:10.3390/antiox13070841)
Supplement: Supplementary file 1 [file antioxidants-13-00841-s001.zip › antioxidants-3069793-supplementary.pdf]

antioxidants

MDPI

Supplementary materials

Antioxidant Properties of Zinc and Copper. Blood Zinc-to Copper-Ratio as a Marker of Cancer Risk BRCA1 Mutation Carriers

Figure S1. Zinc and copper distribution in studied cohort.

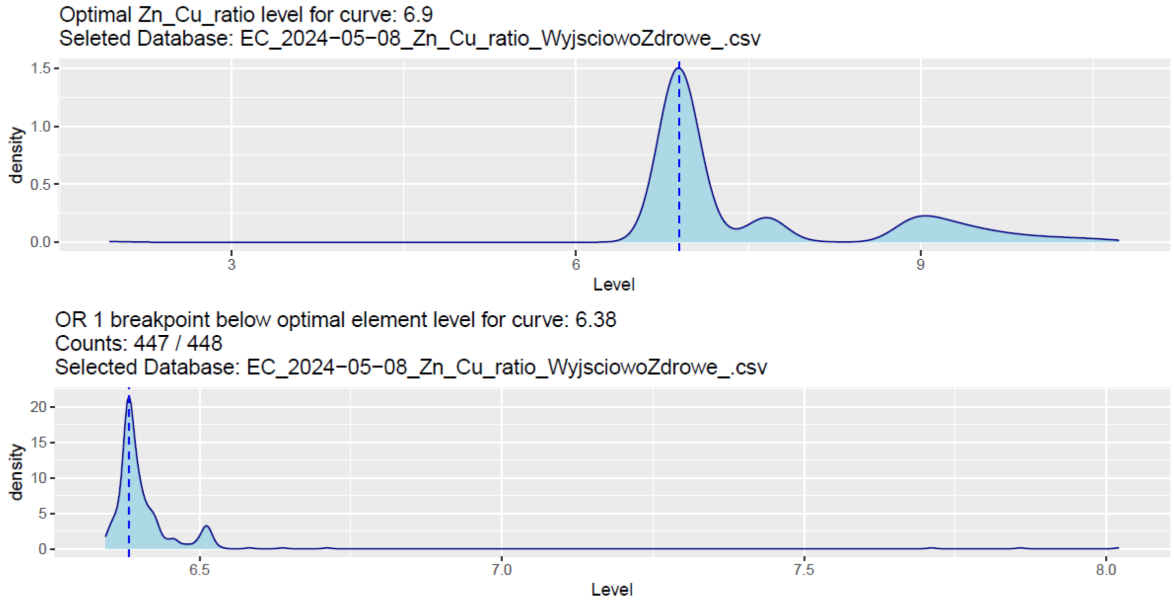

Table S1. Incidence of cancers with different organ localization in initially unaffected BRCA1 mutation carriers according to blood copper levels.

	New Cancer Frequency			New Cancer Univariable COX Regression			New Cancer Multivariable COX Regression		
**Characteristic**	**Overall, N = 919**[1]	**Unaffected, N = 764**[1]	**Cancer, N = 155**[1]	**HR**[2]	**95% CI**[2]	**p-value**	**HR**[2]	**95% CI**[2]	**p-value**
Copper level									
CuT1 **I (reference)** 508.25 - 863.33 (798.35 / 57.16)	303 (33%)	258 (34%)	45 (29%)	—	—		—	—	
CuT2 **II** 863.93 - 971.39 (916.01 / 30.71)	303 (33%)	249 (33%)	54 (35%)	1.25	0.84, 1.86	0.3	1.43	0.95, 2.14	0.084
CuT3 **III** 972.82 - 1,914.47 (1,139.63 / 190.05)	313 (34%)	257 (34%)	56 (36%)	1.21	0.82, 1.79	0.3	1.18	0.79, 1.78	0.4
Age at blood draw									
I (reference) 17.00 - 49.00 (34.57 / 7.61)	725 (79%)	619 (81%)	106 (68%)	—	—		—	—	
II 50.00 - 83.00 (57.27 / 6.02)	194 (21%)	145 (19%)	49 (32%)	1.69	1.20, 2.37	0.002	2.64	1.76, 3.97	<0.001
ANT									
no	457 (50%)	379 (50%)	78 (50%)	—	—		—	—	

	New Cancer Frequency			New Cancer Univariable COX Regression			New Cancer Multivariable COX Regression		
**Characteristic**	**Overall**, N = 919[1]	**Unaffected**, N = 764[1]	**Cancer**, N = 155[1]	**HR**[2]	**95% CI**[2]	**p-value**	**HR**[2]	**95% CI**[2]	**p-value**
yes	462 (50%)	385 (50%)	77 (50%)	0.96	0.70, 1.32	0.8	1.10	0.79, 1.54	0.6
HTZ									
no	663 (72%)	548 (72%)	115 (74%)	—	—		—	—	
yes	256 (28%)	216 (28%)	40 (26%)	0.78	0.54, 1.11	0.2	1.19	0.79, 1.80	0.4
smoker									
no	675 (73%)	563 (74%)	112 (72%)	—	—		—	—	
yes	244 (27%)	201 (26%)	43 (28%)	1.12	0.79, 1.60	0.5	1.16	0.81, 1.66	0.4
adnex									
yes	495 (54%)	432 (57%)	63 (41%)	—	—		—	—	
no	424 (46%)	332 (43%)	92 (59%)	2.08	1.51, 2.87	<0.001	3.25	2.19, 4.81	<0.001
supplements									
no	469 (51%)	395 (52%)	74 (48%)	—	—		—	—	
yes	450 (49%)	369 (48%)	81 (52%)	1.13	0.83, 1.55	0.4	1.21	0.88, 1.67	0.2
diabetes									
no	857 (93%)	714 (93%)	143 (92%)	—	—		—	—	
yes	62 (6.7%)	50 (6.5%)	12 (7.7%)	1.16	0.64, 2.09	0.6	1.02	0.55, 1.90	>0.9
BMI									
< 18.5	54 (5.9%)	44 (5.8%)	10 (6.5%)	1.16	0.60, 2.24	0.7	1.19	0.61, 2.30	0.6
18.5-24.9	540 (59%)	455 (60%)	85 (55%)	—	—		—	—	
25.0-29.9	232 (25%)	190 (25%)	42 (27%)	1.15	0.80, 1.67	0.4	1.12	0.76, 1.65	0.6
≥ 30.0	93 (10%)	75 (9.8%)	18 (12%)	1.35	0.81, 2.25	0.2	1.17	0.67, 2.04	0.6

Table S2. Incidence of breast cancers in initially unaffected BRCA1 mutation carriers according to blood zinc levels.

	New Cancer Frequency			New Cancer Univariable COX Regression			New Cancer Multivariable COX Regression		
**Characteristic**	**Overall**, N = 871[1]	**Unaffected**, N = 764[1]	**Cancer**, N = 107[1]	**HR**[2]	**95% CI**[2]	**p-value**	**HR**[2]	**95% CI**[2]	**p-value**
Cu									

	New Cancer Frequency			New Cancer Univariable COX Regression			New Cancer Multivariable COX Regression		
**Characteristic**	**Overall**, N = 871[1]	**Unaffected**, N = 764[1]	**Cancer**, N = 107[1]	**HR**[2]	**95% CI**[2]	**p-value**	**HR**[2]	**95% CI**[2]	**p-value**
CuT1 **I** (reference) 508.25 - 863.33 (798.35 / 57.16)	288 (33%)	254 (33%)	34 (32%)	—	—		—	—	
CuT2 **II** 863.93 - 971.39 (916.01 / 30.71)	287 (33%)	250 (33%)	37 (35%)	1.11	0.70, 1.77	0.7	1.32	0.82, 2.12	0.3
CuT3 **III** 972.82 - 1,914.47 (1,139.63 / 190.05)	296 (34%)	260 (34%)	36 (34%)	1.01	0.63, 1.61	>0.9	1.09	0.67, 1.77	0.7
age_of_blood_draw									
I (reference) 17.00 - 49.00 (34.48 / 7.67)	698 (80%)	619 (81%)	79 (74%)	—	—		—	—	
II 50.00 - 83.00 (57.09 / 6.09)	173 (20%)	145 (19%)	28 (26%)	1.32	0.86, 2.04	0.2	2.17	1.30, 3.62	0.003
ANT									
no	428 (49%)	379 (50%)	49 (46%)	—	—		—	—	
yes	443 (51%)	385 (50%)	58 (54%)	1.14	0.78, 1.67	0.5	1.22	0.82, 1.83	0.3
HTZ									
no	626 (72%)	548 (72%)	78 (73%)	—	—		—	—	
yes	245 (28%)	216 (28%)	29 (27%)	0.82	0.53, 1.26	0.4	1.23	0.75, 2.03	0.4
smoker									
no	636 (73%)	563 (74%)	73 (68%)	—	—		—	—	
yes	235 (27%)	201 (26%)	34 (32%)	1.37	0.91, 2.05	0.13	1.39	0.92, 2.11	0.11
adnex									
yes	478 (55%)	432 (57%)	46 (43%)	—	—		—	—	
no	393 (45%)	332 (43%)	61 (57%)	1.94	1.32, 2.85	<0.001	2.68	1.65, 4.34	<0.001
supplements									
no	448 (51%)	395 (52%)	53 (50%)	—	—		—	—	
yes	423 (49%)	369 (48%)	54 (50%)	1.07	0.73, 1.56	0.7	1.14	0.78, 1.68	0.5
diabetes									
no	813 (93%)	714 (93%)	99 (93%)	—	—		—	—	
yes	58 (6.7%)	50 (6.5%)	8 (7.5%)	1.11	0.54, 2.29	0.8	1.14	0.54, 2.43	0.7

	New Cancer Frequency			New Cancer Univariable COX Regression			New Cancer Multivariable COX Regression		
**Characteristic**	**Overall**, N = 871[1]	**Unaf-fected**, N = 764[1]	**Cancer**, N = 107[1]	**HR**[2]	**95% CI**[2]	**p-value**	**HR**[2]	**95% CI**[2]	**p-value**
BMI									
< 18.5	54 (6.2%)	44 (5.8%)	10 (9.3%)	1.55	0.80, 3.03	0.2	1.55	0.79, 3.06	0.2
18.5-24.9	517 (59%)	455 (60%)	62 (58%)	—	—		—	—	
25.0-29.9	216 (25%)	190 (25%)	26 (24%)	1.00	0.63, 1.58	>0.9	1.02	0.63, 1.64	>0.9
≥ 30.0	84 (9.6%)	75 (9.8%)	9 (8.4%)	0.95	0.47, 1.91	0.9	0.88	0.41, 1.87	0.7

Table S3. Incidence of ovarian cancers in initially unaffected BRCA1 mutation carriers according to copper ratio values.

	New Cancer Frequency			New Cancer Univariable COX Regression			New Cancer Multivariable COX Regression		
**Characteristic**	**Overall**, N = 791[1]	**Unaffected**, N = 764[1]	**Cancer**, N = 27[1]	**HR**[2]	**95% CI**[2]	**p-value**	**HR**[2]	**95% CI**[2]	**p-value**
Cu									
CuT1 **I (reference)** 508.25 - 863.33 (798.35 / 57.16)	261 (33%)	256 (34%)	5 (19%)	—	—		—	—	
CuT2 **II** 863.93 - 971.39 (916.01 / 30.71)	261 (33%)	250 (33%)	11 (41%)	2.25	0.78, 6.47	0.13	2.86	0.96, 8.52	0.059
CuT3 **III** 972.82 - 1,914.47 (1,139.63 / 190.05)	269 (34%)	258 (34%)	11 (41%)	2.11	0.73, 6.07	0.2	1.46	0.47, 4.52	0.5
age_of_blood_draw									
I (reference) 17.00 - 49.00 (34.37 / 7.69)	635 (80%)	619 (81%)	16 (59%)	—	—		—	—	
II 50.00 - 83.00 (57.34 / 6.23)	156 (20%)	145 (19%)	11 (41%)	2.61	1.21, 5.64	0.015	7.00	2.59, 18.9	<0.001
ANT									
no	395 (50%)	379 (50%)	16 (59%)	—	—		—	—	
yes	396 (50%)	385 (50%)	11 (41%)	0.67	0.31, 1.45	0.3	0.89	0.39, 2.04	0.8
HTZ									
no	571 (72%)	548 (72%)	23 (85%)	—	—		—	—	
yes	220 (28%)	216 (28%)	4 (15%)	0.40	0.14, 1.16	0.092	0.75	0.22, 2.57	0.6
smoker									
no	584 (74%)	563 (74%)	21 (78%)	—	—		—	—	

	New Cancer Frequency			New Cancer Univariable COX Regression			New Cancer Multivariable COX Regression		
**Characteristic**	**Overall**, N = 791[1]	**Unaffected**, N = 764[1]	**Cancer**, N = 27[1]	**HR**[2]	**95% CI**[2]	**p-value**	**HR**[2]	**95% CI**[2]	**p-value**
yes	207 (26%)	201 (26%)	6 (22%)	0.84	0.34, 2.08	0.7	0.97	0.38, 2.48	>0.9
adnex									
yes	436 (55%)	432 (57%)	4 (15%)	—	—		—	—	
no	355 (45%)	332 (43%)	23 (85%)	8.26	2.85, 24.0	<0.001	25.6	7.73, 85.0	<0.001
supplements									
no	403 (51%)	395 (52%)	8 (30%)	—	—		—	—	
yes	388 (49%)	369 (48%)	19 (70%)	2.45	1.07, 5.60	0.033	3.78	1.55, 9.21	0.003
diabetes									
no	738 (93%)	714 (93%)	24 (89%)	—	—		—	—	
yes	53 (6.7%)	50 (6.5%)	3 (11%)	1.73	0.52, 5.73	0.4	0.82	0.21, 3.18	0.8
BMI									
< 18.5	44 (5.6%)	44 (5.8%)	0 (0%)	0.00	0.00, Inf	>0.9	0.00	0.00, Inf	>0.9
18.5-24.9	468 (59%)	455 (60%)	13 (48%)	—	—		—	—	
25.0-29.9	199 (25%)	190 (25%)	9 (33%)	1.64	0.70, 3.83	0.3	1.67	0.66, 4.23	0.3
≥ 30.0	80 (10%)	75 (9.8%)	5 (19%)	2.40	0.86, 6.74	0.10	1.78	0.53, 5.97	0.4

Table S4. Incidence of breast cancers in initially unaffected BRCA1 mutation carriers according to zinc to copper ratio values.

	New Cancer Frequency			New Cancer Univariable COX Regression			New Cancer Multivariable COX Regression		
**Characteristic**	**Overall**, N = 870[1]	**Unaffected**, N = 763[1]	**Cancer**, N = 107[1]	**HR**[2]	**95% CI**[2]	**p-value**	**HR**[2]	**95% CI**[2]	**p-value**
Zn_Cu_ratio									
II (reference): 6.38 - 16.03 (7.46)	522 (60%)	467 (61%)	55 (51%)	—	—		—	—	
I: 0.00 - 6.37 (5.31)	348 (40%)	296 (39%)	52 (49%)	1.38	0.95, 2.02	0.094	1.31	0.90, 1.93	0.2
year_of_birth									
1965	215 (25%)	181 (24%)	34 (32%)	—	—		—	—	
>1985	134 (15%)	129 (17%)	5 (4.7%)	0.29	0.11, 0.75	0.010	0.30	0.08, 1.15	0.079
1965-1975	215 (25%)	188 (25%)	27 (25%)	0.82	0.49, 1.36	0.4	0.77	0.36, 1.65	0.5

	New Cancer Frequency			New Cancer Univariable COX Regression			New Cancer Multivariable COX Regression		
**Characteristic**	**Overall**, N = 870[1]	**Unaf-fected**, N = 763[1]	**Cancer**, N = 107[1]	**HR**[2]	**95% CI**[2]	**p-value**	**HR**[2]	**95% CI**[2]	**p-value**
1975-1985	306 (35%)	265 (35%)	41 (38%)	0.90	0.57, 1.42	0.7	0.93	0.33, 2.65	0.9
age_of_blood_draw									
≤ 40	527 (61%)	471 (62%)	56 (52%)	—	—		—	—	
>50	158 (18%)	135 (18%)	23 (21%)	1.26	0.77, 2.05	0.4	1.21	0.42, 3.52	0.7
40-50	185 (21%)	157 (21%)	28 (26%)	1.43	0.91, 2.25	0.12	1.47	0.67, 3.21	0.3
ANT									
no	427 (49%)	378 (50%)	49 (46%)	—	—		—	—	
yes	443 (51%)	385 (50%)	58 (54%)	1.14	0.78, 1.67	0.5	1.24	0.82, 1.88	0.3
HTZ									
no	625 (72%)	547 (72%)	78 (73%)	—	—		—	—	
yes	245 (28%)	216 (28%)	29 (27%)	0.82	0.53, 1.26	0.4	0.70	0.44, 1.10	0.12
smoker									
Never	494 (57%)	444 (58%)	50 (47%)	—	—		—	—	
Current	200 (23%)	168 (22%)	32 (30%)	1.66	1.06, 2.59	0.025	1.53	0.97, 2.40	0.065
Former	176 (20%)	151 (20%)	25 (23%)	1.41	0.87, 2.27	0.2	1.32	0.81, 2.15	0.3
BMI									
≤ median (23.05)	441 (51%)	385 (50%)	56 (52%)	—	—		—	—	
> median (23.05)	429 (49%)	378 (50%)	51 (48%)	0.91	0.62, 1.33	0.6	0.82	0.54, 1.23	0.3
adnex									
no	392 (45%)	331 (43%)	61 (57%)						
yes	478 (55%)	432 (57%)	46 (43%)						

Table S5. Incidence of ovarian cancers in initially unaffected BRCA1 mutation carriers according to zinc to copper ratio values.

	New Cancer Frequency			New Cancer Univariable COX Regression			New Cancer Multivariable COX Regression		
**Characteristic**	**Overall**, N = 786[1]	**Unaffected**, N = 763[1]	**Cancer**, N = 23[1]	**HR**[2]	**95% CI**[2]	**p-value**	**HR**[2]	**95% CI**[2]	**p-value**
Zn_Cu_ratio_categories_vector									

	New Cancer Frequency			New Cancer Univariable COX Regression			New Cancer Multivariable COX Regression		
**Characteristic**	**Overall**, N = 786[1]	**Unaffected**, N = 763[1]	**Cancer**, N = 23[1]	**HR**[2]	**95% CI**[2]	**p-value**	**HR**[2]	**95% CI**[2]	**p-value**
II (reference): 6.38 - 16.03 (7.47)	477 (61%)	467 (61%)	10 (43%)	—	—		—	—	
I: 0.00 - 6.37 (5.26)	309 (39%)	296 (39%)	13 (57%)	1.94	0.85, 4.43	0.12	1.78	0.77, 4.11	0.2
year_of_birth									
≤ 1965	189 (24%)	181 (24%)	8 (35%)	—	—		—	—	
>1985	129 (16%)	129 (17%)	0 (0%)	0.00	0.00, Inf	>0.9	0.00	0.00, Inf	>0.9
1965-1975	197 (25%)	188 (25%)	9 (39%)	1.12	0.43, 2.90	0.8	0.82	0.09, 7.89	0.9
1975-1985	271 (34%)	265 (35%)	6 (26%)	0.55	0.19, 1.59	0.3	0.22	0.02, 2.73	0.2
age_of_blood_draw									
≤ 40	484 (62%)	471 (62%)	13 (57%)	—	—		—	—	
>50	142 (18%)	135 (18%)	7 (30%)	1.68	0.67, 4.22	0.3	0.40	0.03, 4.87	0.5
40-50	160 (20%)	157 (21%)	3 (13%)	0.69	0.20, 2.41	0.6	0.20	0.04, 0.98	0.046
ANT									
no	392 (50%)	378 (50%)	14 (61%)	—	—		—	—	
yes	394 (50%)	385 (50%)	9 (39%)	0.63	0.27, 1.46	0.3	0.75	0.30, 1.86	0.5
HTZ									
no	567 (72%)	547 (72%)	20 (87%)	—	—		—	—	
yes	219 (28%)	216 (28%)	3 (13%)	0.35	0.10, 1.16	0.087	0.28	0.08, 0.96	0.044
smoker									
Never	454 (58%)	444 (58%)	10 (43%)	—	—		—	—	
Current	172 (22%)	168 (22%)	4 (17%)	1.07	0.33, 3.40	>0.9	1.09	0.34, 3.52	0.9
Former	160 (20%)	151 (20%)	9 (39%)	2.51	1.02, 6.18	0.045	2.54	1.03, 6.28	0.044
BMI_categories									
≤ median (23.05)	394 (50%)	385 (50%)	9 (39%)	—	—		—	—	
> median (23.05)	392 (50%)	378 (50%)	14 (61%)	1.55	0.67, 3.59	0.3	1.13	0.46, 2.76	0.8
adnex									
no	354 (45%)	331 (43%)	23 (100%)						
yes	432 (55%)	432 (57%)	0 (0%)						
